# Adherence to Mediterranean diet and risk of cancer: an updated systematic review and meta‐analysis of observational studies

**DOI:** 10.1002/cam4.539

**Published:** 2015-10-16

**Authors:** Lukas Schwingshackl, Georg Hoffmann

**Affiliations:** ^1^ Department of Nutritional SciencesFaculty of Life SciencesUniversity of ViennaAlthanstraße 14 UZA II, A‐1090ViennaAustria

**Keywords:** Cancer, Mediterranean diet, meta‐analysis

## Abstract

The aim of the present systematic review and meta‐analysis of observational studies was to gain further insight into the effects of adherence to Mediterranean Diet (MD) on overall cancer mortality, incidence of different types of cancer, and cancer mortality risk in cancer survivors. Literature search was performed using the electronic databases PubMed, and EMBASE until 2 July 2015. We included either cohort (for specific tumors only incidence cases were used) or case–control studies. Study specific risk ratios, hazard ratios, and odds ratios (RR/HR/OR) were pooled using a random effect model. The updated review process showed 23 observational studies that were not included in the previous meta‐analysis (total number of studies evaluated: 56 observational studies). An overall population of 1,784,404 subjects was included in the present update. The highest adherence score to an MD was significantly associated with a lower risk of all‐cause cancer mortality (RR: 0.87, 95% CI 0.81–0.93, *I* ^2^ = 84%), colorectal cancer (RR: 0.83, 95% CI 0.76–0.89, *I* ^2^ = 56%), breast cancer (RR: 0.93, 95% CI 0.87–0.99, *I* ^2^=15%), gastric cancer (RR: 0.73, 95% CI 0.55–0.97, *I* ^2^ = 66%), prostate cancer (RR: 0.96, 95% CI 0.92–1.00, *I* ^2^ = 0%), liver cancer (RR: 0.58, 95% CI 0.46–0.73, *I* ^2^ = 0%), head and neck cancer (RR: 0.40, 95% CI 0.24–0.66, *I* ^2^ = 90%), pancreatic cancer (RR: 0.48, 95% CI 0.35–0.66), and respiratory cancer (RR: 0.10, 95% CI 0.01–0.70). No significant association could be observed for esophageal/ovarian/endometrial/and bladder cancer, respectively. Among cancer survivors, the association between the adherence to the highest MD category and risk of cancer mortality, and cancer recurrence was not statistically significant. The updated meta‐analyses confirm a prominent and consistent inverse association provided by adherence to an MD in relation to cancer mortality and risk of several cancer types.

## Introduction

Dietary quality indexes (DASH pattern, and the Healthy Eating Index) are associated with reduced risk of chronic disease 1, 2. There is considerable evidence that the Mediterranean diet (MD) represents a dietary pattern suitable in the prevention of noncommunicable diseases 3. In March 2014 we published a meta‐analysis of observational studies investigating the effects of compliance with an MD on overall cancer risk (incidence and mortality) and different types of cancer 4. Adherence to the highest category of MD was associated with a significant lower risk of overall cancer mortality/incidence as well as the incidence of several cancer types, especially colorectal cancer, aerodigestive cancer (pharyngeal or esophageal cancer), and prostate cancer.

The number of cancer survivors in the United States and Europe is growing rapidly 5, 6. A few prospective cohort studies investigated the association between composition of diet and cancer survival, reporting inconsistent results 7. For example, several studies focused on the evaluation of the relationship between survival and nutrients rather than dietary patterns 7, 8. Due to the high number of studies that have been published since the release of the previous meta‐analysis, it seems reasonable update the original analysis. Due to the new types of cancer that have meanwhile been taken under consideration and because of the growing importance of cancer survivors, we decided not only to reexecute the original search but to expand the previous meta‐analysis including the effects of an MD diet in cancer survivors as an additional research question.

## Methods

The systematic review protocol of the previous meta‐analysis is registered in PROSPERO International Prospective Register of Systematic Reviews (crd.york.ac.uk/prospero/index.asp Identifier: CRD42013004382). The protocol has meanwhile been adapted to the updated version of this analysis.

### Data sources and searches

Queries of literature were performed using the electronic databases PubMed (until 2 July 2015), and EMBASE (until 2 July 2015), with no restrictions to calendar date using the following search terms:

(“Mediterranean diet” OR “Mediterranean” OR “diet” OR “dietary pattern” OR “dietary score” OR “dietary adherence”) AND (“cancer” OR “neoplasm” OR “neoplastic disease” OR “survivors” OR “recurrence”) AND (“prospective” OR “follow‐ up” OR “cohort” OR “longitudinal”). Search terms added for this update are: “survivors”, “recurrence”, and “longitudinal”. The search strategy had no language restrictions.

Moreover, the reference lists from retrieved articles were checked to search for further relevant studies. Literature search was conducted independently by both authors, with disagreements resolved by consensus.

### Study selection

Cohort studies and case–control studies investigating the association between MD and risk of cancer mortality, cancer types; cancer mortality, and cancer recurrence among cancer survivors were included in this update (for differences between the original analysis and the revised version with respect to grouping of clinical outcomes, see “Statistical analysis”).

The previously established statistical analysis plan was revised in order to pool data if outcomes were reported by at least two studies only, since in meta‐analyses published by the Cochrane collaboration, even single studies are presented and discussed in a systematic review context and the forest plots can still be helpful. Although this refers mainly to interventions or clinical trials, well‐designed prospective cohort studies provide important evidence with complementary strength and limitations as well, especially in the context of nutritional sciences 9.

In addition we expanded our meta‐analysis to include cancer survivors from cohort or case–control studies.

As only two studies of the previous meta‐analysis reported overall cancer incidence (with types of cancer not specified) 4 we focused on overall cancer mortality to increase transparency.

### Data extraction and quality assessment

Data extraction and quality assessment was performed as already reported 4.

### Definition: adherence to MD

Ten studies 10, 11, 12, 13, 14, 15, 16, 17, 18, 19 used the MD score provided by Trichopoulou et al. 20, 10 studies 21, 22, 23, 24, 25, 26, 27, 28, 29, 30 used the alternate MD score established by Fung et al. 31, and Whalen et al. 32 modified the score in relation to dairy foods, grains and starches, and alcohol intakes. Cottet et al. 33 decided to use principal component analysis, whereas Tognon et al. 34 and Xie et al. 35 modified the score by Trichopoulou (adding fruit juices and polyunsaturated fatty acids, focusing on whole grains, and excluding poultry). Two case–control studies 36, 37 applied the MD score established by Panagiotakos et al. 38.

Of the 56 observational studies only six studies excluded the alcohol component [14, 18, 39, 40, 41, 42] from the MD score. Four of these studies focused on risk of breast cancer.

For this meta‐analysis, the lowest adherence to MD category was compared with the highest MD category (according to the MD scores by Trichopoulou, Fung, or Panagiotakos; with the exception of four studies that used factor analysis or principal component analysis to define the MD score: [43] hazard ratio: per 1 standard deviation increase), [44] (odds ratio: fourth vs. first tertile), Cottet et al. (odds ratio: third vs. first tertile), Bessaoud et al. 2012 (odds ratio per increment of one standard error). The maximum ranges of the different MD scores are reported in Table 1.

**Table 1:** General study characteristics of included studies (cohort and case–control studies)

Author [Ref no.]	CountryCohort name	Outcome	PopulationFollow‐up (years)	Age at entry	Sex	Components of scoreScore range	Adjustment	Multivariate adjusted	Quality score (max. 9 points) [65]^a^
Buckland et al. 10	EUEPIC	Bladder cancer	477,31211	35–70	M/W	1. ↑ legumes; 2. ↑ cereals; 3. ↑ fruits/nuts; 4. ↑ vegetables; 5. ↑ fish; 6. ↑ MUFA:SFA ratio; 7. ↔ alcohol; 8. ↓ meat and poultry; 9. ↓ dairy productsMD score range: 0–18	Energy intake, smoking status	HR: 0.84 (0.69, 1.03) for third (12–18) versus first tertile (0–6)	8
Cottet et al. 33	FRAECP	Colorectal cancer incidence among cancer survivors	4423	35–75	M/W	Principal component analysis: ↑ olive oil, fruit, vegetables, legumes, lean meat, fish; ↓ coffee, meat, beer, fats, whole grain bread and vegetables, and delicatessen;MD score range: principal component analysis	Age, treatment group, presence of proximal adenomas at inclusion	OR: 0.61 (0.18, 2.07) principal component analysis for third versus first tertile	5
Cuenca‐García et al. 13	USAACLS	Cancer mortality	12,44911.6	20–84	M/W	1. ↑ legumes; 2. ↑ cereals; 3. ↑ fruits/nuts; 4. ↑ vegetables; 5. ↑ fish; 6. ↑ MUFA:SFA ratio; 7. ↔ alcohol; 8. ↓ meat and poultry; 9. ↓ dairy productsMD score range: 0–9	Age, sex, energy intake, and baseline examination year, PA, cardiorespiratory fitness	HR: 1.63 (0.91, 2.92) for fourth versus first quartile	8
George et al. []	USAWHIOS	Endometrial cancer	84,41513,3	50–79	W	1. ↑ legumes; 2. ↑ whole grain products; 3. ↑ fruits; 4. ↑ nuts; 5. ↑ vegetables; 6. ↑ fish; 7. ↑ MUFA:SFA ratio; 8. ↔ alcohol; 9. ↓ red and processed meatsMD score range: 0–9	Age, energy intake, ethnicity, education, MET‐h/week – PA, diabetes, postmenopausal HRT, oral contraceptive use, age at first birth, participant in Observational study, participant in HT trial, participant in DM trial, alcohol, and BMI	HR: 0.98 (0.82, 1.17) for fifth quintile versus first quintile	8
George et al. 24	USAWHIOS	Cancer mortality	63,80512.9	50–79	W	1. ↑ legumes; 2. ↑ whole grain products; 3. ↑ fruits; 4. ↑ nuts; 5. ↑ vegetables; 6. ↑ fish; 7. ↑ MUFA:SFA ratio; 8. ↔ alcohol; 9. ↓ red and processed meatsMD score range: 0–9	Age, energy intake, ethnicity, educational level, marital status, smoking, PA, postmenopausal HRT, BMI, and diabetes status	HR: 0.80 (0.70, 0.92) for fifth quintile versus first quintile	8
Gnagnarella et al. 11	ITACOSMOS	Lung cancer	4,3365.7	≥50	M/W	1. ↑ legumes; 2. ↑ cereals; 3. ↑ fruits/nuts; 4. ↑ vegetables; 5. ↑ fish; 6. ↑ MUFA:SFA ratio; 7. ↔ alcohol; 8. ↓ meat and poultry; 9. ↓ dairy productsMD score range: 0–9	Baseline risk probability and energy intake	HR: 0.10 (0.01, 0.77) for fifth quintile (8–9) versus first quintile (0–1)	6
Harmon et al. 29	USAMultiethnic Cohort	Cancer mortality	215,78213–18	45–75	M/W	1. ↑ legumes; 2. ↑ whole grain products; 3. ↑ fruits; 4. ↑ nuts; 5. ↑ vegetables; 6. ↑ fish; 7. ↑ MUFA:SFA ratio; 8. ↔ alcohol; 9. ↓ red and processed meatsMD score range: 0–9	Age, BMI, diabetes, energy intake, ethnicity, education, marital status, smoking, PA, HRT, alcohol	HR: ♂ 0.81 (0.75, 0.89) ♀ 0.84 (0.76, 0.92) for fifth quintile (6–9) versus first quintile (0–2)	9
Fung et al. 27	USANHS	Colorectal cancer mortality among cancer survivors	120111.2	30–55	W	1. ↑ legumes; 2. ↑ whole grain products; 3. ↑ fruits; 4. ↑ nuts; 5. ↑ vegetables; 6. ↑ fish; 7. ↑ MUFA:SFA ratio; 8. ↔ alcohol; 9. ↓ red and processed meatsMD score range: 0–9	Age, PA, BMI, weight change, cancer grade, chemotherapy, smoking status, energy intake, colon or rectal cancer, stage of disease, and date of colorectal cancer diagnosis	HR: 0.84 (0.50, 1.42) for fifth quintile (median 6) versus first quintile (median 2)	7
Kenfield et al. [28]	USAHPFS	Prostate Cancer mortality among cancer survivors	51,52924	40–75	M	1. ↑ legumes; 2. ↑ whole grain products; 3. ↑ fruits; 4. ↑ nuts; 5. ↑ vegetables; 6. ↑ fish; 7. ↑ MUFA:SFA ratio; 8. ↔ alcohol; 9. ↓ red and processed meatsMD score range: 0–9	Age at diagnosis, time period, time since diagnosis to FFQ, energy intake, BMI, vigorous PA, smoking status, clinical stage, Gleason score, and treatment, race, height, history of diabetes, family history of prostate cancer, multivitamin use, supplement use did not change the effect estimates for lethal prostate cancer and were left out of the final model, parental history of myocardial infarction before age 60 years, blood pressure, and cholesterol	HR: 1.01 (0.75, 1.38) for third (≥6) versus first tertile (≤3)	9
Kim et al. 26	USANHS	Breast cancer mortality among cancer survivors	2729Diagnosis 1978–1998: follow‐up up trough 2004	30–55	W	1. ↑ legumes; 2. ↑ whole grain products; 3. ↑ fruits; 4. ↑ nuts; 5. ↑ vegetables; 6. ↑ fish; 7. ↑ MUFA:SFA ratio; 8. ↔ alcohol; 9. ↓ red and processed meatsMD score range: 0–9	Age, time since diagnosis, alcohol intake, energy, multivitamin use, BMI, weight change, oral contraceptive use, age, smoking status, PA, stage, categories of treatment, age at first birth and parity, menopausal status and postmenopausal hormone use	HR: 1.15 (0.74, 1.77) for fifth quintile versus first quintile	7
Li et al. 22	USANIH‐AARP	Head and neck cancer	494,967≥10	50–70	M/W	1. ↑ legumes; 2. ↑ whole grain products; 3. ↑ fruits; 4. ↑ nuts; 5. ↑ vegetables; 6. ↑ fish; 7. ↑ MUFA:SFA ratio; 8. ↔ alcohol; 9. ↓ red and processed meatsMD score range: 0–9	Age, race, smoking, alcohol intake, education, BMI, PA, usual activity, and total energy intake	HR: ♂ 0.80 (0.35, 1.01) ♀ 0.42 (0.24, 0.74) for the fifth (7–9) versus first quintile (0–2)	9
Li et al. 22	USANIH‐AARP	Hepatocellular carcinoma	494,942≥10	50–70	M/W	1. ↑ legumes; 2. ↑ whole grain products; 3. ↑ fruits; 4. ↑ nuts; 5. ↑ vegetables; 6. ↑ fish; 7. ↑ MUFA:SFA ratio; 8. ↔ alcohol; 9. ↓ red and processed meatsMD score range: 0–9	Age, sex, race, smoking, alcohol intake, education, BMI, diabetes, usual activity throughout the day, PA, and total energy intake	HR: 0.62 (0.47, 0.84) for the fifth (6–9) versus first quintile (0–2)	9
Lopez‐Garcia et al. 21	USANHSHPFS	Cancer mortality	17,4155.8–7.7	M: 40–75W: 30–55	M/W	1. ↑ legumes; 2. ↑ whole grain products; 3. ↑ fruits; 4. ↑ nuts; 5. ↑ vegetables; 6. ↑ fish; 7. ↑ MUFA:SFA ratio; 8. ↔ alcohol; 9. ↓ red and processed meatsMD score range: 0–9	Age, smoking status, BMI, PA, parental history of myocardial infarction before age 65 year, menopausal status and use of HT in women, multivitamin use, and medication use	HR: ♂ 0.88 (0.63, 1.21) ♀ 0.80 (0.48, 1.33) for the fifth versus first quintile	7
Reedy et al. 25	USANIH‐AARP	Cancer mortality	492,82315	50–70	M/W	1. ↑ legumes; 2. ↑ whole grain products; 3. ↑ fruits; 4. ↑ nuts; 5. ↑ vegetables; 6. ↑ fish; 7. ↑ MUFA:SFA ratio; 8. ↔ alcohol; 9. ↓ red and processed meatsMD score range: 0–9	Age, race/ethnicity, education, marital status, PA, smoking, energy intake, BMI, diabetes, and alcohol	HR: ♂ 0.80 (0.77, 0.83) ♀ 0.79 (0.74, 0.84) for the fifth (6–9) versus first quintile (0–2)	9
Tognon et al. 34	SWEVIP	Respiratory cancer	77,1519	30–60	M/W	1. ↑ vegetables and potatoes; 2. ↑ fruit and juices; 3. ↑ whole grain cereals; 4. ↑ fish and fish products; 5 ↑ ratio of MUFA+PUFA to SFA; 6. ↔ alcohol intakes; 7. ↓ meat and meat products; 8. ↓ dairy productsMD score range: 0–8	Energy intake, age, obesity, smoking status, education, and PA	HR: 0.93 (0.83, 1.04) for cut‐off (>4) versus (≤4)	8
Vormund et al. [15]	SUIMONICANRP 1A	Cancer mortality	17,86121.4	25–74	M/W	1. ↑ legumes; 2. ↑ cereals; 3. ↑ fruits/nuts; 4. ↑ vegetables; 5. ↑ fish; 6. ↑ MUFA:SFA ratio; 7. ↔ alcohol; 8. ↓ meat and poultry; 9. ↓ dairy productsMD score range: 0–9	Age, sex and survey wave, marital status, smoking, BMI, region and nationality	HR: 0.97 (0.95, 1.01) per 1‐point increase	7
Xie et al. 35	USANHS	Ovarian cancer	82,94824	30–55	W	1. ↑ legumes; 2. ↑ whole grain products; 3. ↑ cereal fiber; 4. ↑ fruits; 5. ↑ nuts; 6. ↑ vegetables; 7. ↑ fish; 8. ↑ MUFA:SFA ratio; 9. ↔ alcohol; 10. ↓ red and processed meatsMD score range: 0–10	Age, total energy intake, family history of ovarian cancer, tubal ligation, BMI, parity, number of additional pregnancies, oral contraceptive use duration, smoking, menopausal status, type and duration of PMH use, age at menarche, hysterectomy, unilateral oophorectomy, lactose intake, caffeine intake, PA	HR: 0.91 (0.71, 1.18) for the fifth (≥5.5) versus first quintile (≤2.6)	8

Author [Ref no.]	Country	Outcome	Cases/controls	Age	Sex	Components of score	Adjustment	Multiple adjusted	Quality score (max. 9 points)
Castello et al. 18	SPA	Breast cancer	1017/1107	n.d	W	1. ↑ legumes; 2. ↑ whole grain products; 3. ↑ fruits; 4. ↑ nuts; 5. ↑ vegetables; 6. ↑ fish; 7. ↑ MUFA:SFA ratio; 8. ↓ red and processed meatsMD score range: 0–8	Energy intake, alcohol consumption, BMI, PA, smoking, education, history of breast disease other than cancer, family history of BC, age at menarche, age at first delivery and menopausal status	OR: 0.74 (0.46, 1.18) for fourth (7–8) versus first quartile (0–2)	8
Dalvi et al. 17	USA	Endometrial cancer	647/633	35–79	W	1. ↑ legumes; 2. ↑ cereals; 3. ↑ fruits/nuts; 4. ↑ vegetables; 5. ↑ fish; 6. ↑ MUFA:SFA ratio; 7. ↔ alcohol; 8. ↓ meat and poultry; 9. ↓ dairy productsMD score range: 0–9	Age, race/ethnicity, age at menarche, OC use, parity, energy intake, PA, menopausal status, HRT, and BMI	OR: 0.92 (0.59, 1.43) for fifth versus first quintile	7
Filomeno et al. 12	ITA/SUI	Oral and pharyngeal	768/2078	22–79	M/W	1. ↑ legumes; 2. ↑ cereals; 3. ↑ fruits/nuts; 4. ↑ vegetables; 5. ↑ fish; 6. ↑ MUFA:SFA ratio; 7. ↔ alcohol; 8. ↓ meat and poultry; 9. ↓ dairy productsMD score range: 0–9	Sex, age, study center, year of interview, education, tobacco smoking, body mass index, and total energy intake	OR: 0.20 (0.14, 0.28) for fifth (6–9) versus first quintile (0–2)	6
Filomeno et al. 19	ITA/SUI	Endometrial cancer	1411/3368	22–79	M/W	1. ↑ legumes; 2. ↑ cereals; 3. ↑ fruits/nuts; 4. ↑ vegetables; 5. ↑ fish; 6. ↑ MUFA:SFA ratio; 7. ↔ alcohol; 8. ↓ meat and poultry; 9. ↓ dairy productsMD score range: 0–9	Age, study center, year of interview, education, tobacco smoking, body mass index, age at menopause, age at menarche, parity, oral contraceptive use, hormone replacement therapy use, history of hypertension, diabetes and total energy intake	OR: 0.43 (0.34, 0.56) for fifth (7–9) versus first quintile (0–3)	6
Grosso et al. 36	ITA	Colorectal cancer	338/676	65.3	M/W	1. ↑ nonrefined cereals; 2. ↑ potatoes; 3. ↑ fruits; 4. ↑ vegetables; 5. ↑ legumes; 6. ↑ fish; 7. ↑ olive oil; 8. ↓ red and processed meats; 9. ↓ poultry; 10. ↓ full‐fat dairy products; 11. ↔ alcoholMD score range: 0–55	Age and sex	OR: 0.46 (0.28, 0.75) for third versus first tertile	6
Mourouti et al. 37	GRE	Breast cancer	250/250	56	W	1. ↑ nonrefined cereals; 2. ↑ potatoes; 3. ↑ fruits; 4. ↑ vegetables; 5. ↑ legumes; 6. ↑ fish; 7. ↑ olive oil; 8. ↓ red and processed meats; 9. ↓ poultry; 10. ↓ full‐fat dairy products; 11. ↔ alcoholMD score range: 0–55	Age, educational status, BMI, current smoking, PA, family history of breast cancer, age of menarche, age of menopause, and use of HRT	OR: 0.91 (0.86, 0.97) per 1/55 units	6
Pot et al. 14	UK	Breast cancer	610/1891	57	W	1. ↑ legumes; 2. ↑ cereals; 3. ↑ fruits/nuts; 4. ↑ vegetables; 5. ↑ fish; 6. ↑ MUFA:SFA ratio; 7. ↓ meat and poultry; 8. ↓ dairy productsMD score range: 0–8	Age, parity, use of HRT, weight, height, PA, menopausal status, family history of breast cancer, breast feeding and education level	OR: 1.04 (0.70, 1.53) for third versus first tertile	7
Turati et al. 16	ITA/GRE	Hepatocellular carcinoma	518/772	<85	M/W	1. ↑ legumes; 2. ↑ cereals; 3. ↑ fruits/nuts; 4. ↑ vegetables; 5. ↑ fish; 6. ↑ MUFA:SFA ratio; 7. ↔ alcohol; 8. ↓ meat and poultry; 9. ↓ dairy productsMD score range: 0‐9	Center, age, sex, education, BMI, smoking, diabetes, nonalcohol energy intake, and HBsAg, and/or anti‐HCV positivity	OR: 0.51 (0.34, 0.75) for third (≥5) versus first tertile (0–3)	6
Whalen et al. 32	USA	Colorectal cancer	564/1202	30–74	M/W	1. ↑ vegetables; 2. ↑ fruits; 3. ↑ lean meats; 4. ↑ nuts; 5. ↑ fish; 6. ↑ MUFA:SFA ratio; 7. ↓ red and processed meat; 8. ↓ sodium; 9. ↔ dairy products; 10. ↔ grains and starches; 11. ↔ alcoholMD score range: 11–55	Age, sex, family history of colon cancer in a first‐degree relative, regular use of nonsteroidal anti‐inflammatory drugs, BMI, PA, total energy intake, and use of HRT	OR: 0.77 (0.53, 1.11) for fifth versus first quintile	7

↑, high intake; ↓, low intake; ↔, moderate intake; ACLS, aerobics center longitudinal study; BMI, body mass index; CRC, colorectal cancer; COSMOS, Continuous observation of smoking subjects; EPIC, European prospective investigation into cancer and nutrition; HPFS, health professional follow‐up study; HR, hazard ratio; HRT, hormone replacement therapy; MUFA, monounsaturated fat; NRP 1A, national research program 1 A; NHS, Nurses’ Health Study; OR, odds ratio; PA, physical activity; PUFA, polyunsaturated fat; RR, risk ratio; SFA, saturated fat; VIP, Västerbotten Intervention Program; WHIOS, Women's Health Imitative Observational Study.

a Newcatle Ottawa Scale: Selection (max. 4 points), Comparability (max. 1 point), Exposure (max. 3 points).

### Statistical analysis

The meta‐analysis was performed by combining the multivariable adjusted RRs, HR or ORs of the highest compared with the lowest MD adherence category based on random effects model using Der Simonian–Laird method, which incorporated both within and between study variability. To evaluate the weighting of each study, the standard error for the logarithm HR/RR/OR of each study was calculated and regarded as the estimated variance of the logarithm HR/RR/OR, using an inverse variance method [45]. Studies were grouped according to the different clinical outcomes (overall risk of cancer mortality, risk of colorectal cancer/breast cancer/prostate cancer/gastric cancer/head and neck cancer (pharynx, larynx, oral cavity)/esophageal cancer/pancreatic cancer/liver cancer/ovarian cancer/endometrial cancer/respiratory cancer/bladder cancer). The outcome “aerodigestive cancer” used in the original meta‐analysis was replaced by more detailed categories (i.e., head and neck and esophageal). We expanded our previous meta‐analysis investigating the effects (outcomes: cancer‐specific mortality, and cancer recurrence) of adherence to MD in cancer survivors. Subgroup analysis was performed for cohort studies, and case–control studies. Furthermore, subgroup analyses were performed for pre versus postmenopausal status (breast cancer), and breast cancer subtypes (e.g., ER+/PR+ and HER2−, ER+, ER−, HER2+, HER2−). Furthermore, sensitivity analyses were performed for outcomes presented by at least five studies (no merging of cohort and case–control studies was done in the sensitivity analysis) taking into account country of origin, follow‐up time, and quality of studies. Moreover, to investigate possible sources of heterogeneity across studies, we performed a meta‐regression analysis to investigate the effects of various characteristics of studies on the study estimates of RRs. All analyses were conducted using the Review Manager by the Cochrane Collaboration (version 5.3) and Stata 12.0 (Stata‐Corp, College Station, TX).

## Results

### Literature search and study characteristics

The detailed steps of the updated meta‐analysis article search (Fig. S1) and selection process are given as an adapted PRISMA flow diagram [46].

Taken together, 23 additional observational studies (14 cohort studies 10, 11, 13, 15, 21, 22, 23, 24, 25, 26, 27, 29, 30, 35, and nine case–control studies 12, 14, 16, 17, 18, 19, 32, 36, 37) were identified that were not included in the previous meta‐analysis. Two studies were included in the original version of this systematic review, data of these were extracted for the update in a modified form: the cohort by Kenfield et al. 28 provided data on cancer survivors previously not synthesized, and the results by Cottet et al. 33 on cancer recurrence were placed in a new context (i.e., cancer survivors).

General study characteristics are summarized in Table 1. Overall, 35 cohort studies including 1,703,579 subjects (incidence cases; bladder: 1425; breast 15,832; colorectal: 8935; endometrial: 1392; esophageal: 848; gastric: 1382; head and neck: 1868; liver: 509; prostate: 29,806; ovarian: 696; respiratory: 124), and 21 case–control studies with 80,825 subjects met the objectives and were included in the updated meta‐analysis (Supplemental References). The total number of subjects in the included studies was 1,784,404.

One study resulted to be an updated analysis of cancer mortality outcome of a cohort already included in the previous meta‐analysis, so only the most updated study was added to this final analysis 25.

### Main outcomes

Documentations of the different clinical outcomes are distributed as follows: overall risk of cancer mortality was evaluated in 11 cohorts, breast cancer risk in four cohorts and eight case–control studies, colorectal cancer risk in three cohorts and four case–control studies, prostate cancer risk in three cohorts and one case–control study, gastric cancer risk in two cohorts and one case–control study, head and neck cancer in one cohort study and three case–control studies; endometrial cancer in one cohort and two case–control studies, liver cancer, pancreatic cancer, and esophageal cancer in one cohort study and one case–control study, ovarian cancer, bladder cancer, respiratory cancer, in one cohort study, pancreatic cancer in one case–control study, cancer mortality among cancer survivors in three cohort studies, and cancer recurrence among cancer survivors in one cohort study, and cancer‐specific mortality in one cohort study.

**Figure 1:**
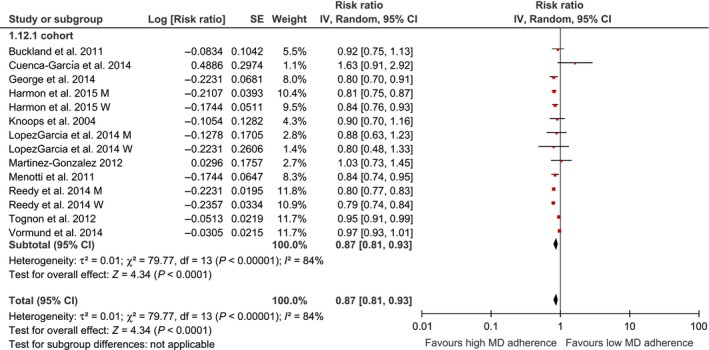
Forest plot showing pooled risk ratios (RRs) with 95% CI for overall cancer mortality risk for eleven cohort studies. I^2^, Inconsistency; MD, Mediterranean Diet; SE, standard error; tau, estimate between study variance.

**Figure 2:**
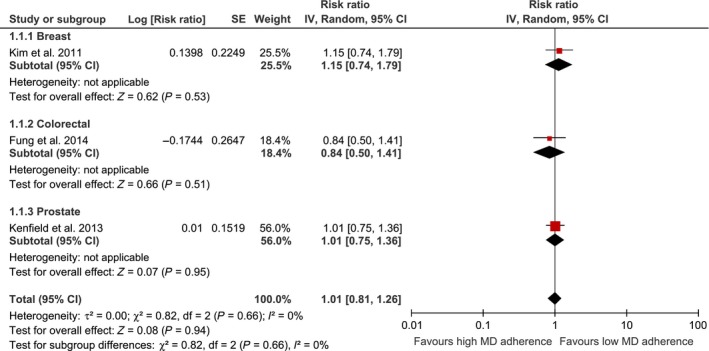
Forest plot showing pooled risk ratios (RRs) with 95% CI for risk of cancer mortality among cancer survivors for three cohort studies. I^2^, Inconsistency; MD, Mediterranean Diet; SE, standard error; tau, estimate between study variance.

Using a random effects model, we found that the highest adherence score to an MD was significantly associated with a lower risk of overall cancer mortality (risk ratio 0.87, 95% confidence interval (CI) 0.81–0.93) (Fig. 1). Among cancer survivors, the association between the adherence to the highest MD category and risk of cancer mortality (RR: 1.01, 95% CI 0.81–1.26), and cancer recurrence (RR: 0.61, 95% CI 0.18–2.07) was not statistically significant (Fig. 2).

With respect to incidence of different types of cancer, enumerative data are summarized in Table 2 and the corresponding forest plots are given as Figures S2–S13.

**Table 2:** Risk ratio/odds ratio associated with the highest adherence to Mediterranean dietary pattern

Outcome	No of studies	Study type	Risk ratio/odds ratio	95% CI	I² (%)
Cancer mortality	11	Cohort	0.87	0.81–0.93	84
Colorectal cancer	7	Combined	0.83	0.76–0.89	56
	3	Cohort	0.84	0.75–0.94	56
	4	Case–control	0.79	0.67–0.93	65
Breast cancer	12	Combined	0.93	0.87–0.99	15
	4	Cohort	0.99	0.89–1.12	33
	8	Case–control	0.90	0.85–0.95	0
Prostate cancer	4	Combined	0.96	0.92–1.00	0
	3	Cohort	0.96	0.92–1.00	0
	1	Case–control	1.03	0.81–1.31	n.a
Gastric cancer	3	Combined	0.73	0.55–0.97	66
	2	Cohort	0.82	0.61–1.10	49
	1	Case–control	0.57	0.45–0.72	n.a
Liver cancer	2	Combined	0.58	0.46–0.73	0
	1	Cohort	0.62	0.47–0.82	n.a
	1	Case–control	0.51	0.34–0.77	n.a
Esophageal cancer	2	Combined	0.49	0.22–1.09	83
	1	Cohort	0.68	0.34–1.36	n.a
	1	Case–control	0.26	0.13–0.52	n.a
Head and neck cancer	4	Combined	0.40	0.24–0.66	90
	1	Cohort	0.61	0.33–1.14	n.a
	3	Case–control	0.32	0.19–0.55	83
Endometrial cancer	3	Combined	0.72	0.40–1.31	94
	1	Cohort	0.98	0.82–1.17	n.a
	2	Case–control	0.61	0.29–1.29	89
Respiratory cancer	1	Cohort	0.10	0.10–0.70	n.a
Bladder cancer	1	Cohort	0.84	0.69–1.02	n.a
Pancreatic cancer	1	Case–control	0.48	0.35–0.66	n.a
Mortality among cancer survivors	3	Cohort	1.01	0.81–1.26	0
Recurrence among cancer survivors	1	Cohort	0.61	0.18–2.07	n.a

n.a, not applicable.

One cohort study investigated the effects of adherence to MD on cancer‐specific mortality. Tognon et al. observed an inverse association between higher adherence to MD and pancreatic cancer mortality, whereas no significant correlation could be detected for breast, colorectal, gastric, prostate, and respiratory cancer mortality, respectively 34.

### Sensitivity analyses

Sensitivity analysis was performed for breast cancer comparing pre versus postmenopausal women. There was a trend for high adherence to MD to be associated with a lower risk of breast cancer in postmenopausal women (RR: 0.95, 95% CI 0.88–1.02) (Fig. S14). Additional sensitivity analyses regarding breast cancer types classified by receptor status yielded significant results comparing the highest versus lowest adherence category to MD only for the ER−/PR+ type (RR: 0.71, 95% CI 0.56–0.89) (Fig. S15).

### Publication bias

The Egger's linear regression tests provided no evidence for a publication bias for overall cancer mortality (*P *= 0.983), and breast cancer (*P *= 0.976), but for colorectal cancer (*P *= 0.096), following comparison of the highest versus lowest adherence to MD category. Funnel plots were only generated when ~10 studies were available for a comparison. The funnel plots for risk of overall cancer mortality as well as risk of breast and colorectal cancer indicate moderate asymmetry, suggesting that publication bias cannot be completely excluded as a factor of influence on the present meta‐analysis (Figs. S16–S18).

### Meta‐regression

To investigate the effects of various study characteristics on the study estimates of the RRs (if at least 5 studies were available), we conducted a meta‐regression analysis (only for cohort studies, since discrepancies compared to case–control are too excessive) by grouping studies according to specific characteristics, that is, sample size, age of the patients, and length of follow‐up. There was a significant inverse association between sample size (*P* < 0.05) and years of age (*P* < 0.05) and risk of cancer mortality, respectively (Figs. S19–S20).

## Discussion

In this updated systematic review and meta‐analysis of observational studies investigating the association between adherence to MD and risk of cancer, findings were pooled from >1.7 million subjects. The main results suggest that adherence to the highest category of an MD is associated with a significant lower risk of overall cancer mortality (by approximately 13%) as well as incidence of colorectal cancer (by 15%), breast cancer (by 7%, no significant lower risk could be observed for cohort studies), gastric cancer (by 27%, no significant lower risk could be observed for cohort studies), prostate cancer (by 4%), liver cancer (by 42%), and head and neck cancer (by 60%, no significant lower risk could be observed for cohort studies). No significant lower risk could be demonstrated with respect to incidence of bladder, ovarian, endometrial, and esophageal cancer. Adherence to an MD has previously been reported to be effective in the primary and secondary prevention of a number of chronic noncommunicable diseases, such as cardiovascular diseases [47], neurodegenerative diseases [48], type 2 diabetes mellitus [49], and neoplastic diseases [50, 51]. We were able to demonstrate an inverse association of MD with respect to overall risk of cancer mortality/incidence and risk of incidence of specific types of cancer in a recently published systematic review 4. We decided to update (and expand) this analysis to synthesize further and additional evidence for a potential inverse association of an MD. Further evidence was granted by the large number of additional observational studies enrolled in this update (+23), resulting in a total of ~1.8 Mio patients.

There are some notable differences when comparing the results of the updated analysis with the first version of 2014 3. In the original analysis, overall cancer mortality data were synthesized together with overall cancer incidence, whenever no information on the specific site of neoplasm was given (yielding a 10% lower risk following adherence to an MD). For reasons of transparency, we decided to focus only on overall cancer mortality in the update. With respect to types of cancer, the positive effects of an MD on colorectal carcinoma demonstrated in the first analysis could be confirmed by the inclusion of new studies. Additional evidence could be found with respect to distinct types of cancer such as liver cancer, or head and neck cancer, which were either not depicted in the original analysis due to lack of corresponding studies or had to be rearranged to fit into the adapted classification of cancer types used for the update. Furthermore, an inverse association could be observed for breast cancer and gastric cancer risk (taking into account the exclusion of Tognon et al., who reported only on cancer‐specific mortality cases). Although it was not always possible to strictly adhere to the cancer categories given in the GLOBOCAN database of the International Agency for the Research on Cancer (http://globocan.iarc.fr), we tried to synthesize the data on various cancer sites by staying as close as possible in line with these definitions. Thus, the cancer type “aerodigestive cancer” used in the original analysis to summarize esophageal/pharyngeal neoplasms has been differentiated into esophageal cancer and head‐and‐neck cancer (laryngeal, pharyngeal, oral cavity). Some data must be interpreted with caution, since the number of observations dealing with these types of cancer are still low (e.g., liver cancer). Likewise, the effects of an MD on breast cancer will remain a matter of debate. The inverse association of an MD dietary pattern after pooling only case–control studies was present in the original analysis and is now further substantiated by three additional studies. However, pooling cohort studies (which are characterized by a higher level of evidence) did not confirm these results (there are no additional cohorts in this update). For future studies, it might be interesting to differentiate between post and premenopausal breast cancer or even to classify breast cancer according to receptor type.

Expanded evidence was expected by synthesizing data on cancer survivors provided by prospective cohort studies. However, we could not find a significant correlation between adherence to an MD and risk of cancer mortality and cancer recurrence. This might be explained by a number of reasons. Cancer survivors are defined as patients with diagnosed cancer, from the time of discovery until death [52]. Fortunately, technical progress in diagnosis and therapy have ensured that the number of cancer survivors is constantly increasing. Thus, it is of paramount importance to establish evidence‐based recommendations for these individuals with respect to lifestyle management. At the same time, the increasing number of cancer survivors results in an increasing heterogeneity of these patients making a one‐size‐fits‐all kind of guideline highly unlikely. For example, different states of diseases are associated with different recommendations (treatment, short‐term or long‐term recovery, stable disease). A number of studies provide evidence that nutrition is an important influencing factor either for tumor progression, recurrence, or survival with most of these reports investigating macronutrient composition or specific nutrients rather than dietary patterns 6, 7. However, there are some studies dealing with the effects of food groups in cancer survivors. A diet rich in fruits, vegetables, whole grains, and fish while at the same time low in red or processed meat as well as refined carbohydrates was associated with decreased mortality rates in breast and colorectal cancer survivors [53, 54, 55]. Most of these food groups fit well within the characteristics of an MD. When breaking down an MD diet into its composing food groups, the item of red wine requires critical balancing. Although consumption of red wine is not expressly requested, a maximum score for adherence to an MD is associated with moderate intake of alcohol in most studies. Alcohol consumption is regarded to be a risk factor in the development of primary tumors of the mouth, pharynx, larynx, esophagus, liver, breast, and colorectal [56]. This will also apply for cancer survivors [57, 58]. In breast cancer survivors, consumption alcohol has been reported to increase mortality risk [59, 60]. On the other hand, a beneficial effect on survival time has been demonstrated in other studies as well [61, 62]. Thus, it is hard to give formulaic advice on red wine (alcohol) consumption for cancer survivors. Most health professionals recommend to avoid any amount of alcohol during cancer treatment, and it is conceivable to modify an MD dietary pattern vie exclusion of red wine either permanently or temporarily.

With respect to protective food groups and their mechanisms of action, an MD is rich in various dietary factors which may affect neoplastic diseases and their outcome in a beneficial manner. New data providing some insight in this topic have recently been published. The results of the largest MD trial, the PREDIMED study including 7447 subjects, showed that the highest category of nuts intake (>3 servings/week) was associated with a 40% risk reduction in cancer mortality when compared to the lowest category [63], whereas the differences observed between consumers of extra virgin olive oil did not attain statistical significance [64]. Insofar, this summarizes the results of a single study and it would be premature to exclude oleic acid or the phenolic content of extra virgin olive oil as a benefactor of an MD.

Although the larger number of studies and patients increased the strength of the updated review, it still has limitations as well. For some cancer sites such as liver cancer, the number of studies is still rather small. In addition, case–control studies may have deficits with respect to measurement and recall bias, while cohort studies have limits regarding validity and reliability of nutritional assessment. In general, incidence and progress of cancer are multifactorial and therefore not only affected by single conditions such as nutrition. In this respect, an MD is a heterogeneous pattern rather than a defined diet. However, the majority of studies in the updated review made use of the MD score established by Trichopoulou et al. 18 or Fung et al. 31 thereby ensuring some degree of homogeneity.

In conclusion, the present systematic review and meta‐analysis provided further evidence that adherence to an MD is associated with lower risk of overall cancer mortality as well as incidence of colorectal, breast, gastric, prostate, liver, and head and neck cancer. If we take into account the number of observations reporting a beneficial effect of an MD in the protection against other chronic diseases as well, it seems reasonable to promote an MD dietary pattern.

## Conflict of Interest

None declared.

## Supporting information


**Figure S1.** Updated flow chart for meta‐analysis article selection process.
**Figure S2.** Forest plot showing pooled risk ratio (RRs) with 95% CI for risk of colorectal cancer for three cohort studies, and four case–control studies.
**Figure S3.** Forest plot showing pooled risk ratio (RRs) with 95% CI for risk of breast cancer for four cohort studies and eight case–control studies.
**Figure S4.** Forest plot showing pooled risk ratio (RRs) with 95% CI for risk of prostate cancer for three cohort studies and one case–control study.
**Figure S5.** Forest plot showing pooled risk ratio (RRs) with 95% CI for risk of gastric cancer for two cohort studies and one case–control study.
**Figure S6.** Forest plot showing pooled risk ratio (RRs) with 95% CI for risk of esophageal cancer for one cohort study and one case–control study.
**Figure S7.** Forest plot showing pooled risk ratio (RRs) with 95% CI for risk of endometrial cancer for one cohort study, and two case–control studies.
**Figure S8.** Forest plot showing pooled risk ratio (RRs) with 95% CI for risk of respiratory cancer for two cohort studies.
**Figure S9.** Forest plot showing pooled risk ratio (RRs) with 95% CI for risk of bladder for one cohort study.
**Figure S10.** Forest plot showing pooled risk ratio (RRs) with 95% CI for risk of pancreatic cancer for one cohort study and one case–control study.
**Figure S11.** Forest plot showing pooled risk ratio (RRs) with 95% CI for risk of liver cancer for one cohort study and one case–control study.
**Figure S12.** Forest plot showing pooled risk ratio (RRs) with 95% CI for risk of head and neck cancer for one cohort study and three case–control studies.
**Figure S13.** Forest plot showing pooled risk ratio (RRs) with 95% CI for risk of ovarian cancer for one cohort study and three case–control studies.
**Figure S14.** Forest plot showing pooled risk ratio (RRs) with 95% CI for risk of breast cancer for pre versus postmenopausal women.
**Figure S15.** Forest plot showing pooled risk ratio (RRs) with 95% CI for risk of breast cancer for breast cancer types.
**Figure S16.** Funnel plot showing study precision against the relative risk effect estimate with 95% CIs for cancer mortality. SE, standard error.
**Figure S17.** Funnel plot showing study precision against the relative risk effect estimate with 95% CIs for colorectal cancer. SE, standard error.
**Figure S18.** Funnel plot showing study precision against the relative risk effect estimate with 95% CIs for breast cancer. SE, standard error.
**Figure S19.** Bubble plot showing the association between sample size and cancer mortality (*P* = 0.045).
**Figure S20.** Bubble plot showing the association between years of age and cancer mortality (*P* = 0.000).
