# Supplementary material for: Adherence to Mediterranean diet and risk of cancer: an updated systematic review and meta‐analysis of observational studies
Source: Cancer Med. 2015 Oct 16;4(12):1933–47. doi: 10.1002/cam4.539 (PMC5123783; doi:10.1002/cam4.539)
Supplement: Supplementary file 1 — Figure S1. Updated flow chart for meta‐analysis article selection process. Figure S2. Forest plot showing pooled risk ratio (RRs) with 95% CI for risk of colorectal cancer for three cohort studies, and four case–control studies. Figure S3. Forest plot showing pooled risk ratio (RRs) with 95% CI for risk of breast cancer for four cohort studies and eight case–control studies. Figure S4. Forest plot showing pooled risk ratio (RRs) with 95% CI for risk of prostate cancer for three cohort studies and one case–control study. Figure S5. Forest plot showing pooled risk ratio (RRs) with 95% CI for risk of gastric cancer for two cohort studies and one case–control study. Figure S6. Forest plot showing pooled risk ratio (RRs) with 95% CI for risk of esophageal cancer for one cohort study and one case–control study. Figure S7. Forest plot showing pooled risk ratio (RRs) with 95% CI for risk of endometrial cancer for one cohort study, and two case–control studies. Figure S8. Forest plot showing pooled risk ratio (RRs) with 95% CI for risk of respiratory cancer for two cohort studies. Figure S9. Forest plot showing pooled risk ratio (RRs) with 95% CI for risk of bladder for one cohort study. Figure S10. Forest plot showing pooled risk ratio (RRs) with 95% CI for risk of pancreatic cancer for one cohort study and one case–control study. Figure S11. Forest plot showing pooled risk ratio (RRs) with 95% CI for risk of liver cancer for one cohort study and one case–control study. Figure S12. Forest plot showing pooled risk ratio (RRs) with 95% CI for risk of head and neck cancer for one cohort study and three case–control studies. Figure S13. Forest plot showing pooled risk ratio (RRs) with 95% CI for risk of ovarian cancer for one cohort study and three case–control studies. Figure S14. Forest plot showing pooled risk ratio (RRs) with 95% CI for risk of breast cancer for pre versus postmenopausal women. Figure S15. Forest plot showing pooled risk ratio (RRs) with 95% CI for risk of [file CAM4-4-1933-s001.doc]

Supporting Information Figure 1: Updated Flow chart for meta-analysis article selection process.

Records identified through database searching: (until 2^nd^ July 2015)
PubMed/EMBASE (n=715)

33 studies included in previous version of review

(n=21 cohort studies)

(n=12 case-control studies)

Additional records identified through other sources
(n = 5)

Records screened after duplicates removed
(n = 465)

Records excluded: title, abstract, not relevant, case-report, cross-sectional study, review, mechanism study

(n=439)

Full-text articles assessed for eligibility
(n = 26)

Full-text articles excluded, with reasons (n =3)

Report the same population (n=1)

Did not evaluate risk of cancer (n=1)

Randomized controlled trial (n=1)

New studies included in qualitative/quantitative synthesis
(n = 23)

Total studies included in quantitative synthesis (meta-analysis)

n=56
(n=35 cohort studies)

(n=21 case-control studies)


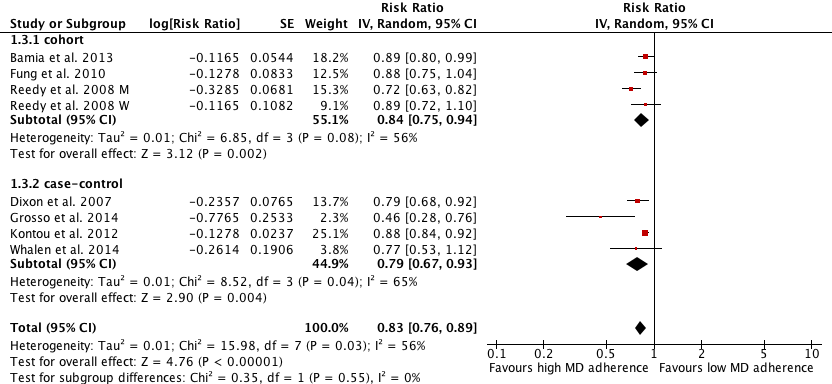


Figure S2. Forest plot showing pooled risk ratio (RRs) with 95% CI for risk of colorectal cancer for three cohort studies, and four case-control studies.


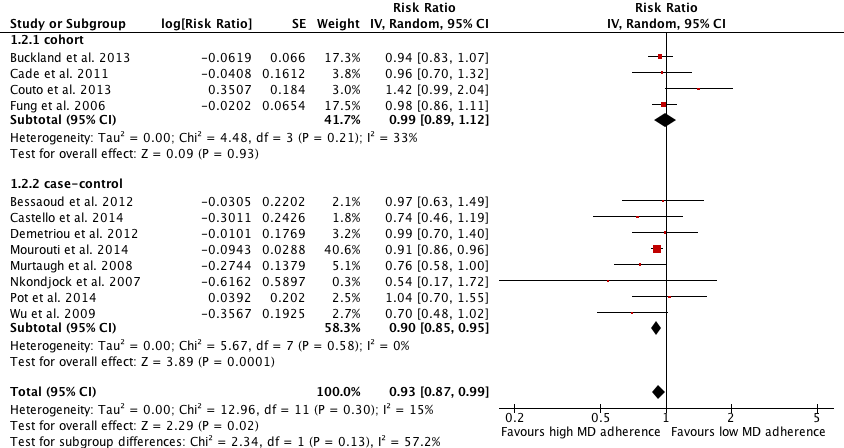


Figure S3. Forest plot showing pooled risk ratio (RRs) with 95% CI for risk of breast cancer for four cohort studies and eight case-control studies.


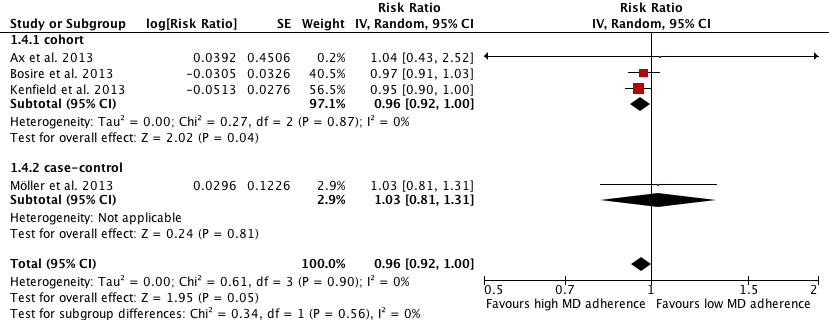


Figure S4. Forest plot showing pooled risk ratio (RRs) with 95% CI for risk of prostate cancer for three cohort studies and one case-control study.


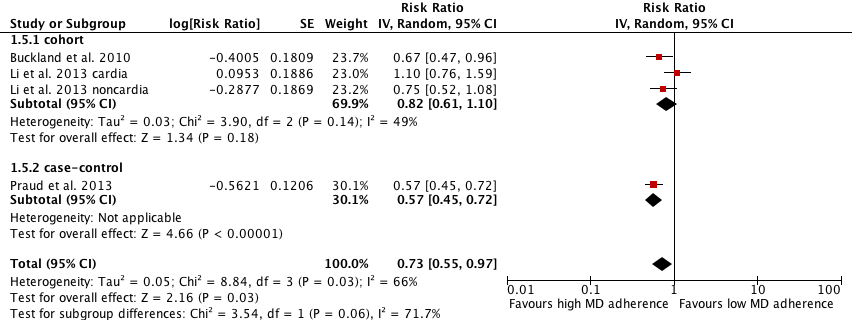


Figure S5. Forest plot showing pooled risk ratio (RRs) with 95% CI for risk of gastric cancer for two cohort studies and one case-control study.


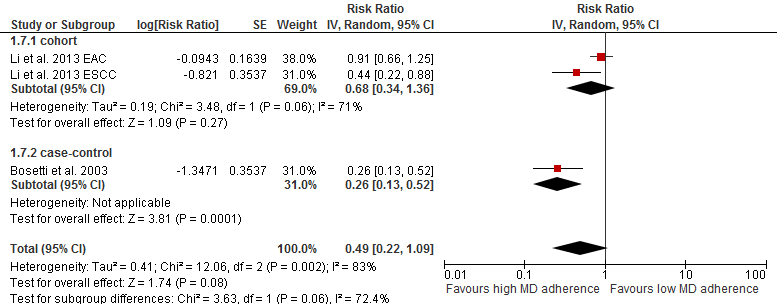


Figure S6. Forest plot showing pooled risk ratio (RRs) with 95% CI for risk of esophageal cancer for one cohort study and one case-control study.


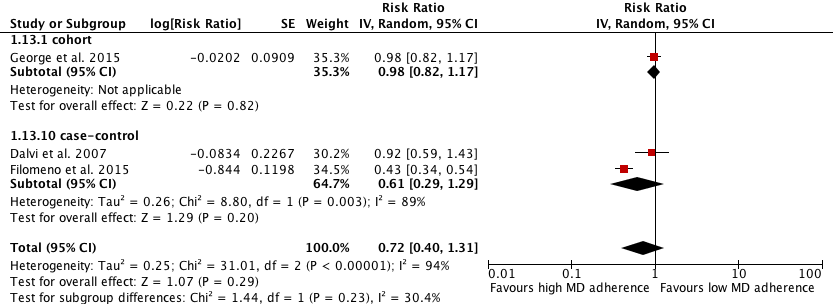


Figure S7. Forest plot showing pooled risk ratio (RRs) with 95% CI for risk of endometrial cancer for one cohort study, and two case-control studies.


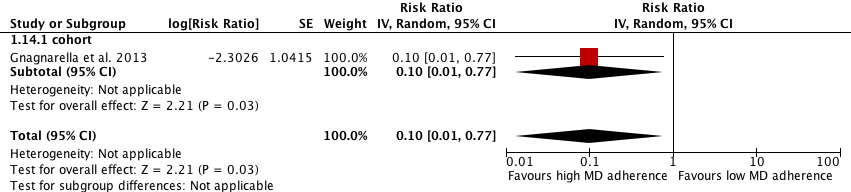


Figure S8. Forest plot showing pooled risk ratio (RRs) with 95% CI for risk of respiratory cancer for two cohort studies.


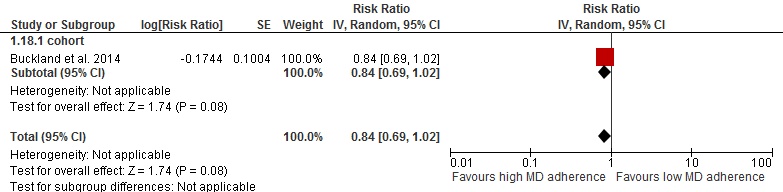


Figure S9. Forest plot showing pooled risk ratio (RRs) with 95% CI for risk of bladder for one cohort study.


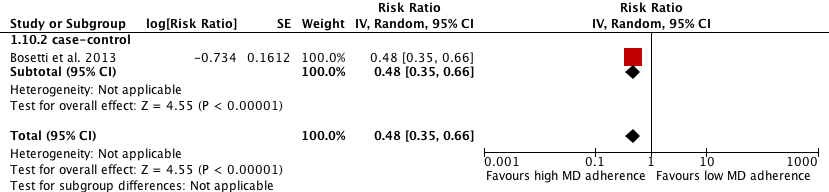


Figure S10. Forest plot showing pooled risk ratio (RRs) with 95% CI for risk of pancreatic cancer for one cohort study and one case-control study.


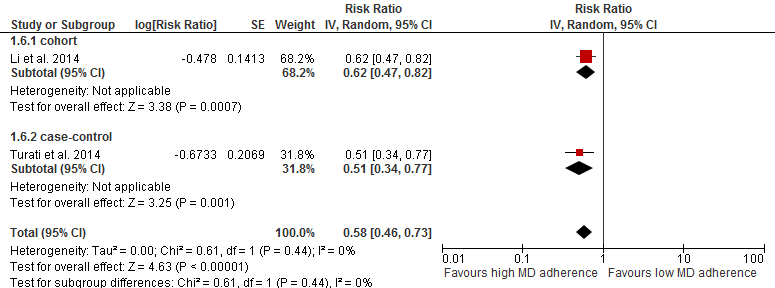


Figure S11. Forest plot showing pooled risk ratio (RRs) with 95% CI for risk of liver cancer for one cohort study and one case-control study.


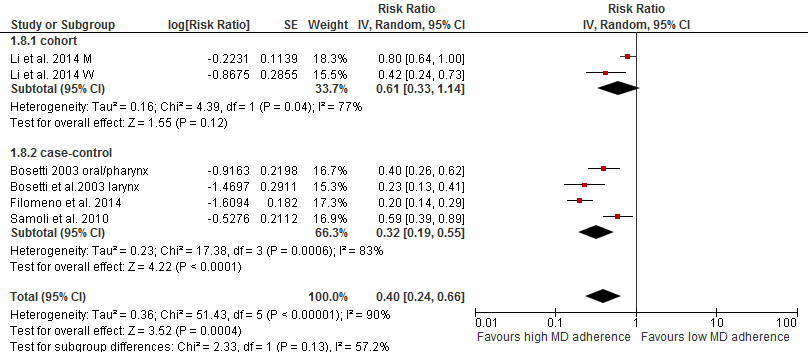


Figure S12. Forest plot showing pooled risk ratio (RRs) with 95% CI for risk of head and neck cancer for one cohort study and three case-control studies.


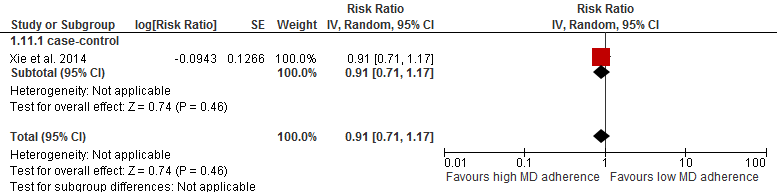


Figure S13. Forest plot showing pooled risk ratio (RRs) with 95% CI for risk of ovarian cancer for one cohort study and three case-control studies.


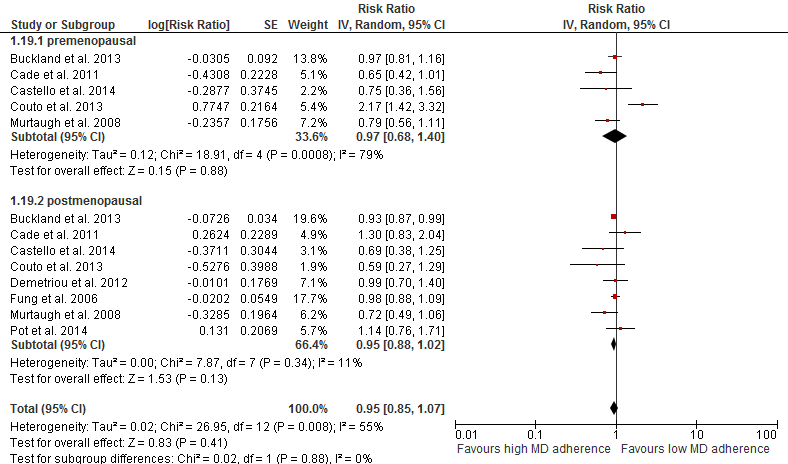


Figure S14. Forest plot showing pooled risk ratio (RRs) with 95% CI for risk of breast cancer for pre vs. postmenopausal women.


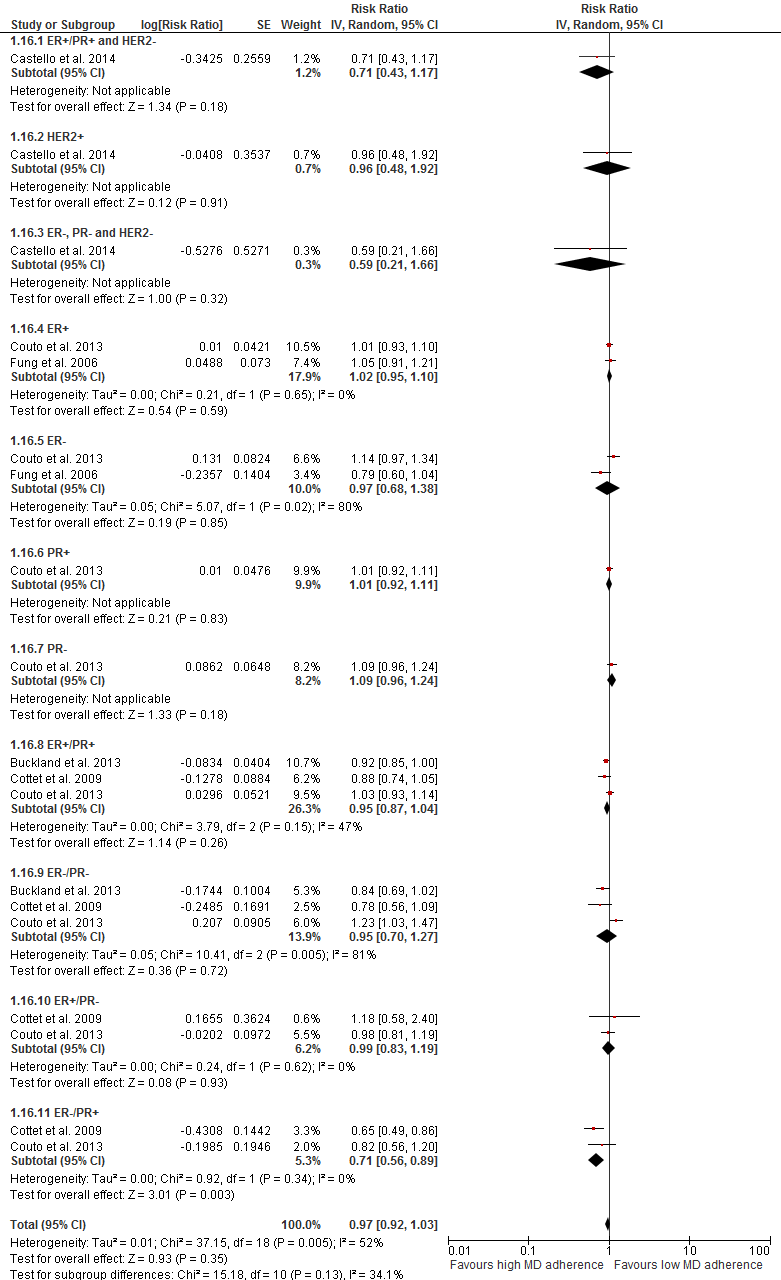


Figure S15. Forest plot showing pooled risk ratio (RRs) with 95% CI for risk of breast cancer for breast cancer types.

Figure S16. Funnel plot showing study precision against the relative risk effect estimate with 95% CIs for cancer mortality. SE = Standard error

Figure S17. Funnel plot showing study precision against the relative risk effect estimate with 95% CIs for colorectal cancer. SE = Standard error

Figure S18. Funnel plot showing study precision against the relative risk effect estimate with 95% CIs for breast cancer; SE = Standard error

Figure S19: Bubble plot showing the association between sample size and cancer mortality (p=0.045)

Figure S20: Bubble plot showing the association between years of age and cancer mortality (p=0.000)

**Supplemental References**

1. Knoops KT, de Groot LC, Kromhout D, Perrin AE, Moreiras-Varela O, Menotti A, van Staveren WA. Mediterranean diet, lifestyle factors, and 10-year mortality in elderly European men and women: the HALE project. JAMA : the journal of the American Medical Association 2004;292:1433-9.

2. Nkondjock A, Ghadirian P. Diet quality and BRCA-associated breast cancer risk. Breast cancer research and treatment 2007;103:361-9.

3. Bamia C, Lagiou P, Buckland G, Grioni S, Agnoli C, Taylor AJ, Dahm CC, Overvad K, Olsen A, Tjonneland A, Cottet V, Boutron-Ruault MC, et al. Mediterranean diet and colorectal cancer risk: results from a European cohort. European journal of epidemiology 2013;28:317-28.

4. Agnoli C, Grioni S, Sieri S, Palli D, Masala G, Sacerdote C, Vineis P, Tumino R, Giurdanella MC, Pala V, Berrino F, Mattiello A, et al. Italian Mediterranean Index and risk of colorectal cancer in the Italian section of the EPIC cohort. International journal of cancer. Journal international du cancer 2013;132:1404-11.

5. Couto E, Boffetta P, Lagiou P, Ferrari P, Buckland G, Overvad K, Dahm CC, Tjonneland A, Olsen A, Clavel-Chapelon F, Boutron-Ruault MC, Cottet V, et al. Mediterranean dietary pattern and cancer risk in the EPIC cohort. British journal of cancer 2011;104:1493-9.

6. Benetou V, Trichopoulou A, Orfanos P, Naska A, Lagiou P, Boffetta P, Trichopoulos D, Greek Ec. Conformity to traditional Mediterranean diet and cancer incidence: the Greek EPIC cohort. British journal of cancer 2008;99:191-5.

7. Buckland G, Travier N, Cottet V, Gonzalez CA, Lujan-Barroso L, Agudo A, Trichopoulou A, Lagiou P, Trichopoulos D, Peeters PH, May A, Bueno-de-Mesquita HB, et al. Adherence to the mediterranean diet and risk of breast cancer in the European prospective investigation into cancer and nutrition cohort study. International journal of cancer. Journal international du cancer 2013;132:2918-27.

8. Trichopoulou A, Bamia C, Lagiou P, Trichopoulos D. Conformity to traditional Mediterranean diet and breast cancer risk in the Greek EPIC (European Prospective Investigation into Cancer and Nutrition) cohort. The American journal of clinical nutrition 2010;92:620-5.

9. Cottet V, Touvier M, Fournier A, Touillaud MS, Lafay L, Clavel-Chapelon F, Boutron-Ruault MC. Postmenopausal breast cancer risk and dietary patterns in the E3N-EPIC prospective cohort study. American journal of epidemiology 2009;170:1257-67.

10. Buckland G, Agudo A, Travier N, Huerta JM, Cirera L, Tormo MJ, Navarro C, Chirlaque MD, Moreno-Iribas C, Ardanaz E, Barricarte A, Etxeberria J, et al. Adherence to the Mediterranean diet reduces mortality in the Spanish cohort of the European Prospective Investigation into Cancer and Nutrition (EPIC-Spain). The British journal of nutrition 2011;106:1581-91.

11. Couto E, Sandin S, Lof M, Ursin G, Adami HO, Weiderpass E. Mediterranean dietary pattern and risk of breast cancer. PloS one 2013;8:e55374.

12. Martinez-Gonzalez MA, Guillen-Grima F, De Irala J, Ruiz-Canela M, Bes-Rastrollo M, Beunza JJ, Lopez del Burgo C, Toledo E, Carlos S, Sanchez-Villegas A. The Mediterranean diet is associated with a reduction in premature mortality among middle-aged adults. The Journal of nutrition 2012;142:1672-8.

13. Bosetti C, Turati F, Pont AD, Ferraroni M, Polesel J, Negri E, Serraino D, Talamini R, Vecchia CL, Zeegers MP. The role of Mediterranean diet on the risk of pancreatic cancer. British journal of cancer 2013;109:1360-6.

14. Möller E, Galeone C, Andersson T, Bellocco R, Adami HO, Andrén O, Grönberg H, La Vecchia C, Mucci L, K; B. Mediterranean Diet Score and prostate cancer risk in a Swedish population-based case-control study. Journal of Nutritional Sciences 2013;2:1-13.

15. Murtaugh MA, Sweeney C, Giuliano AR, Herrick JS, Hines L, Byers T, Baumgartner KB, Slattery ML. Diet patterns and breast cancer risk in Hispanic and non-Hispanic white women: the Four-Corners Breast Cancer Study. The American journal of clinical nutrition 2008;87:978-84.

16. Buckland G, Agudo A, Lujan L, Jakszyn P, Bueno-de-Mesquita HB, Palli D, Boeing H, Carneiro F, Krogh V, Sacerdote C, Tumino R, Panico S, et al. Adherence to a Mediterranean diet and risk of gastric adenocarcinoma within the European Prospective Investigation into Cancer and Nutrition (EPIC) cohort study. The American journal of clinical nutrition 2010;91:381-90.

17. Cade JE, Taylor EF, Burley VJ, Greenwood DC. Does the Mediterranean dietary pattern or the Healthy Diet Index influence the risk of breast cancer in a large British cohort of women? European journal of clinical nutrition 2011;65:920-8.

18. Lagiou P, Trichopoulos D, Sandin S, Lagiou A, Mucci L, Wolk A, Weiderpass E, Adami HO. Mediterranean dietary pattern and mortality among young women: a cohort study in Sweden. The British journal of nutrition 2006;96:384-92.

19. Dixon LB, Subar AF, Peters U, Weissfeld JL, Bresalier RS, Risch A, Schatzkin A, Hayes RB. Adherence to the USDA Food Guide, DASH Eating Plan, and Mediterranean dietary pattern reduces risk of colorectal adenoma. The Journal of nutrition 2007;137:2443-50.

20. Wu AH, Yu MC, Tseng CC, Stanczyk FZ, Pike MC. Dietary patterns and breast cancer risk in Asian American women. The American journal of clinical nutrition 2009;89:1145-54.

21. Fung TT, Hu FB, McCullough ML, Newby PK, Willett WC, Holmes MD. Diet quality is associated with the risk of estrogen receptor-negative breast cancer in postmenopausal women. The Journal of nutrition 2006;136:466-72.

22. Fung TT, Hu FB, Wu K, Chiuve SE, Fuchs CS, Giovannucci E. The Mediterranean and Dietary Approaches to Stop Hypertension (DASH) diets and colorectal cancer. The American journal of clinical nutrition 2010;92:1429-35.

23. Li WQ, Park Y, Wu JW, Ren JS, Goldstein AM, Taylor PR, Hollenbeck AR, Freedman ND, Abnet CC. Index-based Dietary Patterns and Risk of Esophageal and Gastric Cancer in a Large Cohort Study. Clinical gastroenterology and hepatology : the official clinical practice journal of the American Gastroenterological Association 2013;11:1130-6 e2.

24. Reedy J, Mitrou PN, Krebs-Smith SM, Wirfalt E, Flood A, Kipnis V, Leitzmann M, Mouw T, Hollenbeck A, Schatzkin A, Subar AF. Index-based dietary patterns and risk of colorectal cancer: the NIH-AARP Diet and Health Study. American journal of epidemiology 2008;168:38-48.

25. Mitrou PN, Kipnis V, Thiebaut AC, Reedy J, Subar AF, Wirfalt E, Flood A, Mouw T, Hollenbeck AR, Leitzmann MF, Schatzkin A. Mediterranean dietary pattern and prediction of all-cause mortality in a US population: results from the NIH-AARP Diet and Health Study. Archives of internal medicine 2007;167:2461-8.

26. Samoli E, Lagiou A, Nikolopoulos E, Lagogiannis G, Barbouni A, Lefantzis D, Trichopoulos D, Brennan P, Lagiou P. Mediterranean diet and upper aerodigestive tract cancer: the Greek segment of the Alcohol-Related Cancers and Genetic Susceptibility in Europe study. The British journal of nutrition 2010;104:1369-74.

27. Bosire C, Stampfer MJ, Subar AF, Park Y, Kirkpatrick SI, Chiuve SE, Hollenbeck AR, Reedy J. Index-based dietary patterns and the risk of prostate cancer in the NIH-AARP diet and health study. American journal of epidemiology 2013;177:504-13.

28. Bessaoud F, Tretarre B, Daures JP, Gerber M. Identification of dietary patterns using two statistical approaches and their association with breast cancer risk: a case-control study in Southern France. Annals of epidemiology 2012;22:499-510.

29. Demetriou CA, Hadjisavvas A, Loizidou MA, Loucaides G, Neophytou I, Sieri S, Kakouri E, Middleton N, Vineis P, Kyriacou K. The mediterranean dietary pattern and breast cancer risk in Greek-Cypriot women: a case-control study. BMC cancer 2012;12:113.

30. Bosetti C, Gallus S, Trichopoulou A, Talamini R, Franceschi S, Negri E, La Vecchia C. Influence of the Mediterranean diet on the risk of cancers of the upper aerodigestive tract. Cancer epidemiology, biomarkers & prevention : a publication of the American Association for Cancer Research, cosponsored by the American Society of Preventive Oncology 2003;12:1091-4.

31. Kontou N, Psaltopoulou T, Soupos N, Polychronopoulos E, Xinopoulos D, Linos A, Panagiotakos DB. Metabolic syndrome and colorectal cancer: the protective role of Mediterranean diet--a case-control study. Angiology 2012;63:390-6.
